# How financial mechanisms can incentivize provision of ecosystem services from land restoration: A systematic review protocol

**DOI:** 10.1371/journal.pone.0289120

**Published:** 2023-07-24

**Authors:** Vasilis Grigoriadis, Elizabeth Gold, George Hutchinson, Lynn J. Frewer, Paul Brereton, Darragh Flannery, Kenneth A. Byrne, John Garvey

**Affiliations:** 1 Institute for Global Food Security, Queen’s University Belfast, Belfast, United Kingdom; 2 Gibson Institute, Queen’s University Belfast, Belfast, United Kingdom; 3 School of Natural and Environmental Sciences, Newcastle University, Newcastle Upon Tyne, United Kingdom; 4 Kemmy Business School, University of Limerick, Limerick, Ireland; 5 Department of Biological Sciences, School of Natural Sciences, University of Limerick, Limerick, Ireland; CSIR National Environmental Engineering Research Institute (CSIR-NEERI), INDIA

## Abstract

The current food chain both contributes to, and is affected by, climate change. While GHG emissions and emissions to water and soil are a problem for the whole food chain, the majority of such emissions and the major solutions to them can be found in the farming and land use sector. The farming system needs to reduce its greenhouse-gas emissions and adapt its supply chain to cope with climate change. A broad variety of payment tools have been proposed to motivate farmers and landowners to take certain actions to reduce greenhouse gas emissions and encourage the protection or restoration of natural resources. The protocol described here (OSF preregistration https://doi.org/10.17605/OSF.IO/STGQ6) outlines the methodology for a systematic review to explore how financial mechanisms such as green bonds can provide incentives to agri-food sector to support environmental sustainability and ecosystem service delivery through land-use change. Our primary research question is: how do financial mechanisms incentivize land restoration? Studies will be categorized according to the types of financial mechanisms, their characteristics, methods of land restoration and their impact on mitigating agri-food footprint. The results are expected to increase our understanding about the design of financing tools currently used to accelerate nature restoration. Moreover, they will inform us about the effectiveness of deploying such tools on rural communities, food companies and landowners.

## Introduction

The twin anthropogenic pressures of climate change [1] and biodiversity loss [2] present the planet with far reaching challenges that need to be addressed. During 2007–2016 total net greenhouse gas emissions from Agriculture, Forestry and other land use accounted for 23% of total net anthropogenic greenhouse gas emissions [3]. While GHG emissions and emissions to water and soil are a problem for the whole food chain, the majority of such emissions arise in the farming sector. Reducing these emissions and associated environmental impact will require changes in land management that promote large scale carbon sequestration and other ecosystem services which are also found in the farming and land use sector.

Restoration policies have failed to be implemented in many geographical areas; for example, Ireland is characterised by low forest cover (11.4%) relative to the mean of EU for 2020 (39.8%; [4]). Moreover, forests that have been planted in the past years are at risk of becoming a net source of carbon back to the atmosphere due to an unbalanced age structure and a reduction in afforestation rates in recent years [5].

Few new ideas on how to change the status quo are emerging and the range of public finance solutions are relatively limited in the context of economic and political pressures on governments. In addition, individual agencies, landowners, or interest groups are unlikely to have the available capital to perform large scale and long-term afforestation or forest management projects with the potential to produce tangible changes. The financial sanctions of failing to meet the climate targets enshrined in recent international climate agreements demonstrate the direct economic costs involved and motivate innovative approaches to resolving this challenge [6]. Conversely, delivering on large scale native afforestation has the potential to bend the curve towards meeting the primary climate goals of carbon neutrality by 2050. Innovative and successful implementation has the potential to achieve this while restoring ecologies and providing new opportunities for rural communities and the agri-food industry to thrive.

The island of Ireland provides a framing context for the current review. Ireland’s inability to meet afforestation targets will impede the State’s ability to achieve the targets set by the Paris Climate Change Agreement and the European Commission, and lead to financial sanctions [6, 7]. In the South, the target is to increase Ireland’s forest cover to 18% of Ireland’s land area; and ensure that at least 30% of the area afforested comprises broadleaves, including native woodland. Planting targets of 8,000 ha / year have been committed to as part of the 2019 Climate Action Plan, while in Northern Ireland, policy aims to plant 9,000 ha over the next decade, compared to the recent average of 200 ha / year [8, 9].

Forestry funding schemes in the North and South are broadly similar. Current incentives are designed to fund the planting costs associated with afforestation and a tax-free premium paid to farmers over the course of the first fifteen years of tree growth [10]. Existing public funding is not having the desired impact and the barriers to undertaking land-use exist in the form of competition from existing and long-standing agricultural enterprise and food production, complexity in the interacting issues around biodiversity restoration and commercial imperatives from afforestation as well as linked challenges around forestry licensing. The potential availability of private or mixed funding using financial instruments that are linked to afforestation and land-use change may provide the Irish agri-food industry with a way to have a meaningful environmental impact and one that has a local and observable environmental and social benefit. Novel financial mechanisms can be used in conjunction with other adaptations to their core business–ensuring the sector contributes to national environmental priorities and maintain a positive economic impact on rural communities. This provides the motivation for the current review and will help to provide generalisable insights into the set of financial mechanisms that have been used to accelerate environmental restoration.

More specifically, the primary research question is: How and can financial mechanisms ptimizede land restoration to improve environmental sustainability and ecosystem service delivery? To frame our research question, we use the following terms:

- Financial instruments: “Any contract that gives rise to a financial asset of one entity and a financial liability or equity instrument of another entity” [11].
- Land restoration is the “process of ecological restoration of a site to a natural landscape and habitat, safe for humans, wildlife, and plant communities” [12]. Moreover, ecological restoration is the “intentional activity that initiates or accelerates the recovery of an ecosystem with respect to its health, integrity and sustainability” [13].
- Environmental sustainability is defined as “responsible interaction with the environment to avoid depletion or degradation of natural resources and allow for long-term environmental quality. The practice of environmental sustainability helps to ensure that the needs of today’s population are met without jeopardizing the ability of future generations to meet their needs” [14].
- Ecosystem services are the “benefits people obtain from ecosystems. These include provisioning services such as food, water, timber, and fiber; regulating services that affect climate, floods, disease, wastes, and water quality; cultural services that provide recreational, aesthetic, and spiritual benefits; and supporting services such as soil formation, photosynthesis, and nutrient cycling” [15].

Moreover, the PICO (Population, Intervention, Comparison and Outcomes) framework is a quantitative analysis tool used to identify different elements of evidence for a systematic review [16]. The key PICO elements for our question can be formulated as follows:

- Population: Land restoration
- Intervention: Financial mechanisms
- Comparator: no intervention
- Outcomes: Environmental improvement

The secondary research questions for the systematic review are:

- What is the geographic distribution and spatial resolution (local, national, continental, global) of the studies?
- What are the financial tools and their characteristics (e.g., duration, transaction size, risks, interest rate) in the studies?
- What kinds of land restoration (e.g., reforestation, wetland restoration) are considered in the studies?
- Who are the payers and/or beneficiaries/co-beneficiaries (e.g., oil or water companies, forest landowners, government/citizens) of the land restoration?
- How is environmental improvement measured in the studies (e.g., carbon mitigation, biodiversity loss)?
- What ecosystem services are considered in the studies (e.g., flood risk reduction, water quality, wildfire risk reduction)?
- What community development implications are considered in the studies (e.g., farmers’ incomes, reduced depopulation, improvements to public services such as health /education)?

## Materials and methods

This review protocol follows the guidelines of PRISMA-P (Preferred reporting items for systematic review and meta-analysis protocols) which support the planning and documentation of review methods and act as a guard against subjective decision making during review conduct [17]. Moreover, the protocol was based on the PRISMA-P checklist which has 17 items considered to be essential and minimum components of a systematic review (S1 File).

### Bibliographic databases and data management

A structured search was conducted in 3 databases: Scopus, Web of Science and Agricultural & Environmental Science Collection. These databases have been selected based on their relevance to the field of study and their comprehensiveness. The search engine of Google scholar will be used (200 first results) to identify academic or grey literature not captured by the search of bibliographic databases.

The database searches will be done under the existing subscriptions of the organizations of the authors. Search results will be downloaded and catalogued in the EndNote 20 version.

### Search string scoping

Searching of bibliographic databases were conducted using a search string. The search string was formulated based on the research question and the PICO elements. Keywords for each PICO element were proposed by our research team which consists of experts in the following areas: environmental and ecological economics, risk management and finance, land use change on soil carbon stocks and greenhouse gas emissions and food supply chains (the members of our research team are also authors of this protocol). These keywords have been tested and ptimized by conducting a scoping exercise in Scopus, following the CEE guidelines [18]. Narrower and wider search strings were trialed during scoping to ensure an appropriate balance of specificity (reducing the number of irrelevant studies) and sensitivity (ensuring all relevant studies are identified). The study we targeted as most relevant and should be identified in each search is the work of [19]. This study designs an environmental impact bond for the restoration of a wetland area in Louisiana.

### Search string formulation

The search string uses the Boolean operators OR and AND to identify literature that combines terms for the three components: financial mechanisms, land restoration, and, environmental sustainability and ecosystem service delivery (Table 1). Within each bibliographic database, the search string was adapted to the format required for that database but will use the same terms and search fields (Title, Abstract and Keywords). S2 File shows the search string details for each database.

**Table 1:** Search string to be used for bibliographic databases (the three components will be combined with the Boolean operator AND).

Financial Mechanisms	Land restoration	Environmental sustainability and ecosystem service delivery
“sustainable finance” OR “environmental impact bond*” OR “securitization” OR “real option*” OR “finance option*” OR “municipal bond*” OR “PFP” OR “pay for performance” OR “pay-for-performance” OR “pay-for-success” OR “pay for success” OR “green bond*” OR “resilience bond*” OR “sustainability bond*” OR “green bond*” OR “climate-aligned bond*” OR “climate aligned bond*” OR “forest bond*” OR “catastrophe bond*” OR “cat bond*” OR “risk pool*” OR “agricultural grants” OR “payments for environmental services” OR “payments for ecosystem services”	“land restoration” OR “environmental restoration” OR “rehabilitation” OR “hydrologic restoration” OR “*forest*” OR “tree planting” OR “woodland*” OR “wetland*” OR “peatland*” OR “bog*” OR “carbon sequestration” OR “carbon sink*” OR “GHG sink*” OR “greenhouse gas sink*” OR “mangroves” OR “coral reef*”	“environmental sustainability” OR “climate change mitigation” OR “carbon mitigation” OR “carbon reduction” OR “GHG mitigation” OR “GHG reduction” OR “greenhouse gas mitigation” OR “greenhouse gas reduction” OR “global warming mitigation” OR “biodiversity” OR “eutrophication” OR “ecosystem service*” OR “resilience” OR “flood protection” OR “waste treatment” OR “waste management” OR “cultural” OR “aesthetic” OR “soil protection” Or “habitat protection”

## Article screening and study eligibility criteria

### Screening strategy

Two stages of screening will be conducted. The first stage will assess the relevance of each article based on title and abstract. Articles that clearly meet the exclusion criteria will be excluded, and, articles that meet the eligibility criteria (or where there is uncertainty) will be included for screening at stage two. Stage two will assess the relevance of each article included from the first stage on the basis of full text. Again, articles that meet any of the exclusion criteria will be excluded, and articles that meet all of the eligibility criteria will be included. Where there is uncertainty, articles will be included and marked for a second opinion.

Screening will be performed using drop-down menus listing exclusion criteria. This will allow us to record the reasons for which each article is excluded. This process will be conducted using the software Endnote 20. This software will also be used to remove duplicate articles when the same article is returned by more than one bibliographic database or search engine.

### Inter-reviewer reliability

At each stage of the screening process, the articles will be screened by two reviewers to assess consistency of decisions between reviewers. The Cohen’s kappa [20] will be estimated to test the degree of agreement between the two reviewers. Similar to [21] we will use a threshold of 0.6. If the kappa result is over 0.6, this will be considered an acceptable level of agreement for inter-reviewer reliability and any disagreements will be discussed and resolved. If the kappa result is less than 0.6 the eligibility and exclusion criteria will be discussed (and amended if necessary) by the review team to improve consistency of interpretation between reviewers. The process will be repeated at each stage until a kappa result over 0.6 is achieved.

### Eligibility criteria

The articles will be included or excluded at screening according to the following PICO criteria:

*Population*: *Land restoration*
  - Land restoration is key to restore degraded land to stop biodiversity loss, and reinstate ecosystem services for human well-being [22]. In this review, we include documents where restoration is the focus of study, e.g., reforestation. On the other side, we exclude documents that are interested in the maintenance and protection of the natural and habitat environment. Many of those papers belong to the scientific area of forest and agroforest management.
  - Research conducted in all geographical location will be included.
*Intervention*: *Financial mechanisms*
  - This study will review financial mechanisms including green bonds, options contracts, and, payments for performance or environmental services. On the other side, it will exclude auctions and trading systems, and, exclusively public funding (e.g., through national taxes).
*Outcomes*: *Environmental improvement*
  - This study is interested in documents that aim to improve environmental sustainability and/or ecosystem services.

Other eligibility criteria that will be considered are:

- Only academic literature in English from 1989 to present will be included.
- Study type: Books and review papers will be excluded from the coding process.
- Theoretical papers: Papers that are purely policy focused will be excluded from the coding process.

### Study validity assessment

This review will generate a narrative synthesis of existing approaches to researching and conceptualizing financial mechanisms to incentivize the land restoration. All studies meeting inclusion criteria, including those that are not peer-reviewed and regardless of their risk of bias, will be included in the synthesis. Whether a study has been peer-reviewed will be noted in the data extraction spreadsheet.

Moreover, follow-up techniques such as reference tracking and citation searching will be applied to final studies to locate any additional references [23].

### Data extraction

Information on each of the studies will be collected in an Excel spreadsheet made available to all study authors. The spreadsheet will provide places to record the following information, if available, for each article under review. To ensure the study is repeatable, EG will extract data from all articles and VG will extract data from every 10^th^ article. Agreement will be assessed using the same criteria used for article screening. If the kappa result is over 0.6, this will be considered an acceptable level of agreement for inter-reviewer reliability and any disagreements will be discussed and resolved. If the kappa result is less than 0.6 we will clarify the data extraction approaches and then the process will be repeated until a kappa result over 0.6 is achieved.

#### Data categorization

Information on each of the studies will be collected in an Excel spreadsheet made available to all study authors. The spreadsheet will provide places to record the following information, if available, for each article under review:

- Full study citation
- Peer-reviewed? (Yes, No)
- Continent(s) where the study is based
- Country(ies) where the study is based
- Economic mechanism (e.g., green bonds)
- Duration of mechanism (in years)
- Transaction size
- Information about risks
- Global or local risks
- Interest rate
- Land restoration (e.g., reforestation, wetland restoration)
- Payers and/or beneficials (e.g., oil or water companies, forest landowners, government/citizens)
- Environmental improvement measurement (e.g., carbon mitigation, biodiversity loss)
- Ecosystem service (e.g., flood risk reduction, water quality, wildfire risk reduction)
- Community development implications (e.g., farmers’ incomes, reduced depopulation, improvements to public services such as health /education)

### Data synthesis

This systematic review will not conduct any quantitative, statistical synthesis of data (meta-analysis). Thematic coding will be used to identify and interpret patterns in the data relevant to the research questions. A systematic narrative synthesis will be provided with the information presented in text and tables to summarize and explain the characteristics and findings of the included studies. All studies meeting inclusion criteria, regardless of their risk of bias, will be included in the synthesis.

#### Risk of publication bias

The authors have no conflicts of interest. To reduce reviewer selection bias, two reviewers participate in the study screening and data extraction reaching a standard degree of agreement using Cohen’s kappa.

## Conclusions

This protocol will try to find evidence on how private financial mechanisms can support land restoration as a significant way to improve environmental sustainability and ecosystem service delivery. The expected results of this study are intended to enhance our understanding of the design of funding mechanisms used to accelerate nature restoration. Furthermore, they will provide valuable insights into the efficacy of implementing these tools on rural communities, food companies, and landowners.

One main limitation of this study is the focus on land restoration as a purposeful action of recuperation without including evidence related to maintenance and protection of the natural environment. The reasoning behind this is driven by the necessity to discover suitable methodologies tailored to the unique circumstances of Ireland, which is characterized by a relatively low forest cover (11.4%) and a substantial presence of agricultural activities. However, future literature reviews could investigate the private financial mechanisms that target not only the land restoration but also the maintenance and protection of the natural environment.

Another limitation is that our study mainly focuses on evidence derived from literature. Future research could thoroughly investigate the green mechanisms reported by finance organizations and their applications in the field. However, considering that the use of green private finance tools is relatively recent, evidence regarding their effectiveness may require more time to become available.

## Supporting information

S1 File PRISMA-P checklist3. (DOCX)

S2 File Search string details for each database. (DOCX)
