# Supplementary material for: How financial mechanisms can incentivize provision of ecosystem services from land restoration: A systematic review protocol
Source: PLoS One. 2023 Jul 24;18(7):e0289120. doi: 10.1371/journal.pone.0289120 (PMC10365305; doi:10.1371/journal.pone.0289120)
Supplement: S2 File — (DOCX) [file pone.0289120.s002.docx]

**S2 File. Search string details for each database.**

| **Database** | **Query string** | **Filters** | **No of documents** |
| --- | --- | --- | --- |
| **Scopus** | TITLE-ABS-KEY (("sustainable finance" OR "environmental impact bond*" OR "securitization" OR "real option*" OR "municipal bond*" OR "PFP" OR "pay for performance" OR "pay-for-performance" OR "pay-for-success" OR "pay for success" OR "green bond*" OR "resilience bond*" OR "sustainability bond*" OR "green bond*" OR "climate-aligned bond*" OR "climate aligned bond*" OR "forest bond*" OR "catastrophe bond*" OR "cat bond*" OR "finance option*" OR "risk pool*" OR "agricultural grants" OR "payments for environmental services" OR "payments for ecosystem services" ) AND ( "land restoration" OR "environmental restoration" OR "hydrologic restoration" OR "*forest*" OR "tree planting" OR "woodland*" OR "wetland*" OR "peatland*" OR "bog*" OR "carbon sequestration" OR "carbon sink*" OR "GHG sink*" OR "greenhouse gas sink*" OR "mangroves" OR "coral reef*" OR "rehabilitation" ) AND ( "environmental sustainability" OR "climate change mitigation" OR "carbon mitigation" OR "carbon reduction" OR "GHG mitigation" OR "GHG reduction" OR "greenhouse gas mitigation" OR "greenhouse gas reduction" OR "global warming mitigation" OR "biodiversity" OR "eutrophication" OR "ecosystem service*" OR "resilience" OR "flood protection" OR "waste treatment" OR "waste management" OR "cultural" OR "aesthetic" OR "soil protection" OR "habitat protection")) | Exclude books and book chapters, Include only English | 907 |
| **Web of Science** | ((TS=("sustainable finance" OR "environmental impact bond*" OR "securitization" OR "real option*" OR "municipal bond*" OR "PFP" OR "pay for performance" OR "pay-for-performance" OR "pay-for-success" OR "pay for success" OR "green bond*" OR "resilience bond*" OR "sustainability bond*" OR "green bond*" OR "climate-aligned bond*" OR "climate aligned bond*" OR "forest bond*" OR "catastrophe bond*" OR "cat bond*" OR "finance option*" OR "risk pool*" OR "agricultural grants" OR "payments for environmental services" OR "payments for ecosystem services" )) AND TS=("land restoration" OR "environmental restoration" OR "hydrologic restoration" OR "*forest*" OR "tree planting" OR "woodland*" OR "wetland*" OR "peatland*" OR "bog*" OR "carbon sequestration" OR "carbon sink*" OR "GHG sink*" OR "greenhouse gas sink*" OR "mangroves" OR "coral reef*" OR "rehabilitation" )) AND TS=( "environmental sustainability" OR "climate change mitigation" OR "carbon mitigation" OR "carbon reduction" OR "GHG mitigation" OR "GHG reduction" OR "greenhouse gas mitigation" OR "greenhouse gas reduction" OR "global warming mitigation" OR "biodiversity" OR "eutrophication" OR "ecosystem service*" OR "resilience" OR "flood protection" OR "waste treatment" OR "waste management" OR "cultural" OR "aesthetic" OR "soil protection" OR "habitat protection”) | Exclude books | 646 |
| **Agricultural & Environmental Science Collection** | (("sustainable finance" OR "environmental impact bond*" OR "securitization" OR "real option*" OR "municipal bond*" OR "PFP" OR "pay for performance" OR "pay-for-performance" OR "pay-for-success" OR "pay for success" OR "green bond*" OR "resilience bond*" OR "sustainability bond*" OR "green bond*" OR "climate-aligned bond*" OR "climate aligned bond*" OR "forest bond*" OR "catastrophe bond*" OR "cat bond*" OR "finance option*" OR "risk pool*" OR "agricultural grants" OR "payments for environmental services" OR "payments for ecosystem services" ) AND ( "land restoration" OR "environmental restoration" OR "hydrologic restoration" OR "*forest*" OR "tree planting" OR "woodland*" OR "wetland*" OR "peatland*" OR "bog*" OR "carbon sequestration" OR "carbon sink*" OR "GHG sink*" OR "greenhouse gas sink*" OR "mangroves" OR "coral reef*" OR "rehabilitation") AND ("environmental sustainability" OR "climate change mitigation" OR "carbon mitigation" OR "carbon reduction" OR "GHG mitigation" OR "GHG reduction" OR "greenhouse gas mitigation" OR "greenhouse gas reduction" OR "global warming mitigation" OR "biodiversity" OR "eutrophication" OR "ecosystem service*" OR "resilience" OR "flood protection" OR "waste treatment" OR "waste management" OR "cultural" OR "aesthetic" OR "soil protection" OR "habitat protection")) | Search only in titles, exclude books and book chapters, include only English | 80 |
| **Google Scholar** | ("financial mechanism" OR "financial instruments" OR "environmental impact bond" OR "green bond") AND ("land restoration" OR "environmental restoration" OR "*forest*" OR "woodland") AND ("environmental sustainability" OR "ecosystem service")) | 1987-2022 | First 200 results |
